# New record for the genus *Platymessa* Mello-Leitão, 1941 in Colombia, with the description of a new species (Opiliones, Cosmetidae)

**DOI:** 10.3897/zookeys.665.11371

**Published:** 2017-04-03

**Authors:** Conchita A. Pinzón-M., Victor R. Townsend, Jr., Neis Martínez-H.

**Affiliations:** 1 Departamento de Biología, Universidad del Atlántico Km 7 vía a Puerto Colombia, Barranquilla, Colombia; 2 Department of Biology, Virginia Wesleyan College 1584 Wesleyan Drive, Norfolk, Virginia 23502, USA

**Keywords:** *Platymessa victoriae* sp. n., Cesar Department, Colombia

## Abstract

The genus *Platymessa* Mello-Leitão, 1941 is represented by two species in the Andes of Colombia: *P. h-inscriptum* Mello-Leitão, 1941 and *P. ectroxantha* Mello-Leitão, 1941. *Platymessa victoriae* Pinzón-M. & Townsend, **sp. n.** is described on the basis of somatic morphological characters and the structure of the penis. The placement of this new species in the genus *Platymessa* is based upon multiple characters including the outline of dorsal scutum, the presence of a blunt spine on coxa IV, having short and strong legs with femora III and IV having five longitudinal rows of small tubercles, the shape of the basitarsomeres of male leg I, the distribution and relative sizes of the marginal setae on the ventral plate of the penis, and the morphology of the chelicerae and cheliceral sockets. In contrast to other members of the genus, *P. victoriae* has a pair of triangular tubercles on scutal area III, lacks paired paramedian tubercles on scutal area V, and does not have a ladder mask color pattern on the dorsal scutum. The description of this species expands the distribution of the genus to north of the Oriental Cordillera in the Cesar Department of Colombia.

## Introduction

The family Cosmetidae Koch, 1839 is distributed from the southern U.S. to Argentina and with over 700 species, it is the second largest family of harvestman in the suborder Laniatores (Medrano and Kury 2016). Cosmetid harvestmen also are one of the best represented families of laniatorean harvestmen in the Neotropics (Kury and Pinto-da-Rocha 2007). The major distinguishing feature of cosmetid harvestmen is the lateral compression of the tibiae of the pedipalps that partially cover the chelicerae at rest (Pinto-da-Rocha 2011). The genus *Platymessa* Mello-Leitão, 1941 was originally diagnosed using characters based upon the Roewerian system, including the morphology of tarsus I and the ornamentation of the dorsal scutum. Two species were described from the Colombian Andes, *P. h-inscriptum* Mello-Leitão, 1941 and *P. nigrolimbata* Mello-Leitão, 1941. A third species, *P. transversalis*, was described by Roewer (1963) but has been subsequently transferred to the genus *Chusgonobius* Roewer, 1952 by Medrano and Kury (2016). Recently, Medrano and Kury (2016) used several characters to redescribe *Platymessa* including those based upon the shape of the dorsal scutum, the presence of a pair of paramedian granules on scutal area V, armature of coxa IV, and the morphology of the legs. Medrano and Kury (2016) also redescribed *P. h-inscriptum* as the type species of the genus and considered *P. nigrolimbata* as a junior synonym of *P. h-inscriptum*. In addition, Medrano and Kury (2016) transferred *Brachylibitia ectroxantha* Mello-Leitão, 1941 to the genus *Platymessa* proposing the new combination of *Platymessa ectroxantha*. In this paper, we propose the recognition of a third Colombian species in the genus *Platymessa* Mello-Leitão, 1941 on the basis of several characters including those based upon penis morphology. This is the first record of the genus for northern Colombia.

## Methods

We examined 15 males and 28 females collected from the type locality (see below). Specimens were photographed with a Leica MC-120 HD digital camera attached to a Leica S8AP0 stereomicroscope and then processed with the software CombineZP. The illustrations were made with the aid of stereomicroscope with a camera lucida, Wild type 308700, Heerbrugg Switzerland. The illustrations of the penis were made from photographs taken with an optical Leica CME microscope and the software Inkscape version 0.91. The map was made with ArcGIS.

The shape of the dorsal scutum was described using the system proposed by Kury et al. (2007). We used the nomenclature for the macrosetae of the ventral plate of the penis that was proposed by Kury and Villarreal (2015), but refer to MS C3 as D1 and D1 as D2 (following system discussed in Medrano and Kury 2016). Terminology used for the description of the fields of microsetae on the ventral plate of the penis is based upon Kury (2016). Color names and codes follow Ridgway (1912). All of the measurements are in mm. Abbreviations: Cx; coxa, CW: carapace width, CL: carapace length, DSW: dorsal scutal width, Fe: femur, DSL: dorsal scutal length, ICN: Instituto de Ciencias Naturales, Mt: metatarsus, MS: macrosetae, Pa: patella, Ta: tarsus, Ti: tibia, Tr: trochanter, TBL: total body length, UA: Universidad del Atlántico, UNAL: Universidad Nacional de Colombia.

## Taxonomy

### Cosmetidae C.L. Koch, 1839

#### Platymessa

Taxon classificationAnimaliaOpilionesCosmetidae

Mello-Leitão, 1941

Platymessa Mello-Leitão, 1941: 167; Roewer 1963: 52; Kury 2003: 81 (type species Platymessa H-inscripta Mello-Leitão, 1941, by original designation).
Platimessa [incorrect original spelling]: Mello-Leitão 1941: 167.
Brachylibitia Mello-Leitão, 1941: 166; Kury 2003: 38 [junior subjective synonym of Cynorta C.L. Koch, 1839 by Goodnight & Goodnight (1953: 38); synonymy disclaimed by Kury (2003: 38)]; Medrano & Kury 2016: 54-57 [Junior synonym of Platymessa Mello-Leitão 1941; type species: Brachylibitia ectroxantha Mello-Leitão, 1941, by original designation].

#### Platymessa victoriae

Taxon classificationAnimaliaOpilionesCosmetidae

Pinzón-M. & Townsend sp. n.

http://zoobank.org/43268445-8D44-481B-A1DC-6AEAEACFBDD0

##### Diagnosis.

This species differs from *P. h-inscriptum* and *P. ectroxantha* by the presence of multiple blunt tubercles in scutal area I, paired triangular tubercles in scutal area III, the absence of a pair of larger paramedian granules on dorsal scutal area V, lacking a ladder mask color pattern on the dorsal scutum, and instead having a V-shaped color pattern on the cephalic groove and a transverse line in the groove between areas III-IV on the dorsal scutum.

##### Type locality.

Colombia, Cesar Departament, Municipality of Manaure Balcón del Cesar, páramo de Sabana Rubia, 10°22'8.6"N; 72°53'33.6"W, 3200 m of elevation, 29 October 2015. C. Pinzón-M.

##### Type material.

Holotype (ICN-AO-1030). Adult male preserved in 96% ethanol, penis in a microvial with 70% ethanol. Original label: “CO, Cesar, Manaure, Páramo de Sabana Rubia, 10°22'8.6"N; 72°53'33.6"W, 29 October 2015. The holotype, allotype and paratypes will be deposited in the Collection of the Arachnida at the Instituto de Ciencias Naturales (ICN-AO), National University of Colombia (ICN). Collector: C. Pinzón-M.

##### Paratypes.

21 individuals (10 ♂ ICN-AO-1032, ICN-AO-1033, ICN-AO-1034, ICN-AO-1035, ICN-AO-1036, ICN-AO-1037, ICN-AO-1038, ICN-AO-1039, ICN-AO-1040, ICN-AO-1041 and 11♀ ICN-AO-1042, ICN-AO-1043, ICN-AO-1044, ICN-AO-1045, ICN-AO-1046), Colombia, Cesar, Manaure, October 29, 2015. Collected with the holotype. 22 individuals (5 ♂ and 17 ♀), Colombia, Cesar, Manaure, March 12, 2016. Same data as the holotype.

##### Etymology.

The new species is named to honor the memory of María Victoria Pinzón M.

##### Description of the male holotype.

**Figure 1.**
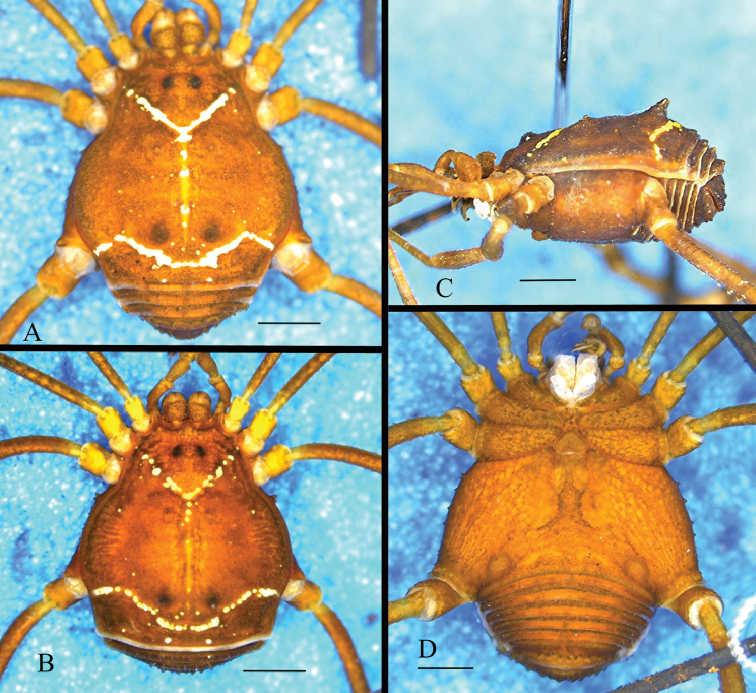
*Platymessa victoriae* sp. n. **A** Habitus, dorsal view (male holotype, ICN-AO-1030) **B** Habitus, dorsal view (female allotype, ICN-AO-1031) **C** Habitus lateral view (male holotype) **D** Ventral view (male holotype). Scale bar: 1mm.

**Figure 2.**
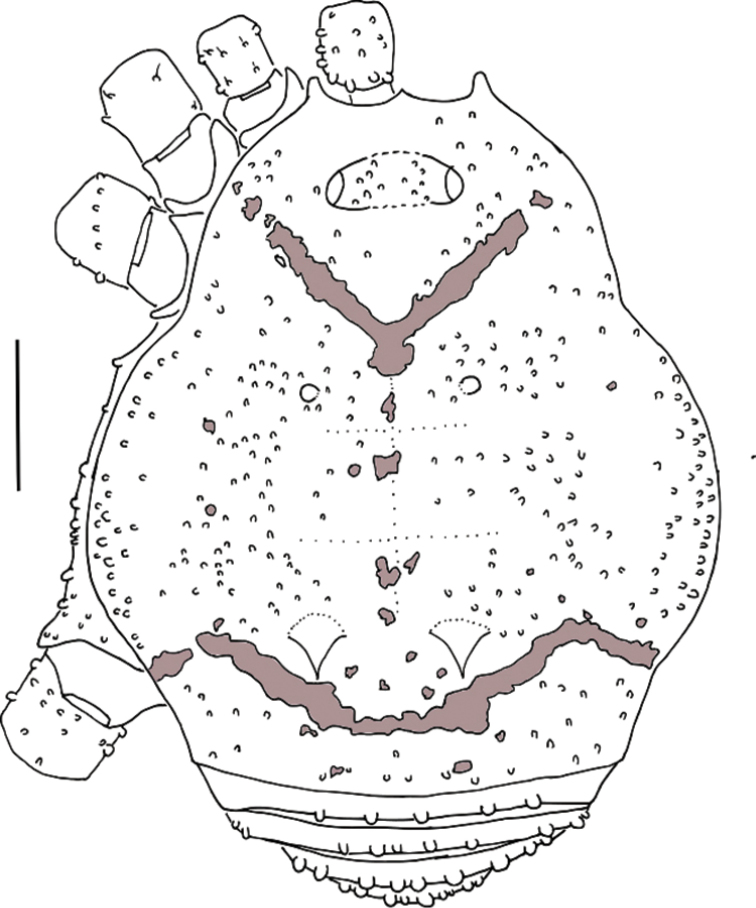
*Platymessa victoriae* sp. n. (male holotype, ICN-AO-1030). Habitus, dorsal view. Scale bar: 1 mm.

**Figure 3.**
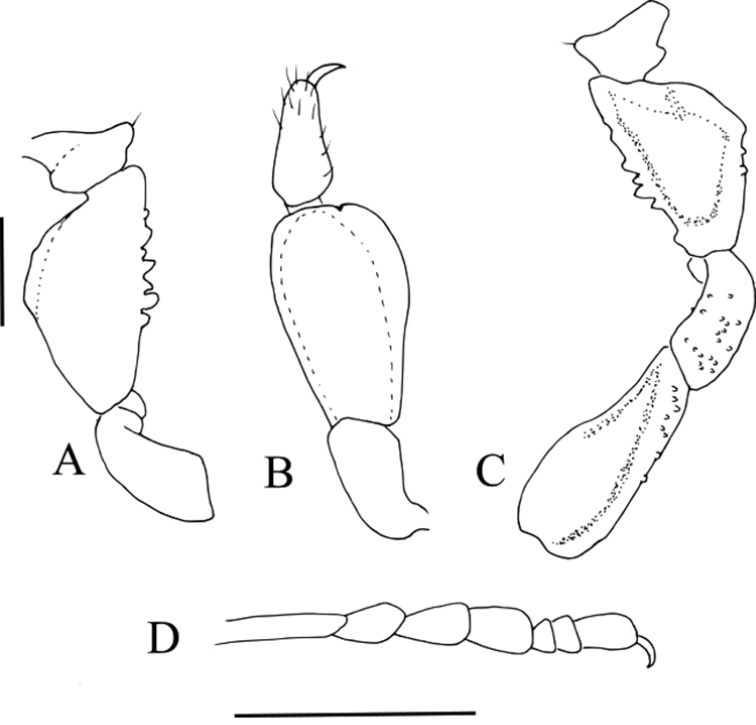
*Platymessa victoriae* sp. n. (male paratype, ICN-AO-1032) **A** Left pedipalp, Tro, Fe and Pa in ectal view **B** Same, Ti and Ta in dorsal view **C** Same in mesal view **D** Left basitarsus I in prolateral view. Scale bar: 1 mm.

**Figure 4.**
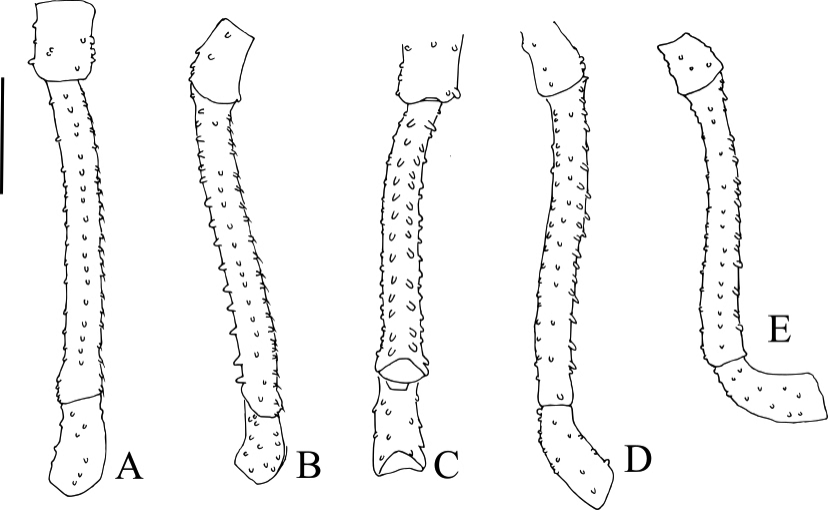
*Platymessa victoriae* sp. n. (male paratype, ICN-AO-1032) Left leg IV. **A** Fe in dorsal view **B** Same, in prolateral view **C** Same, in ventral view **D** Same, in retrolateral view **E** Left leg III, Fe in retrolateral view. Scale bar: 1mm.

**Figure 5.**
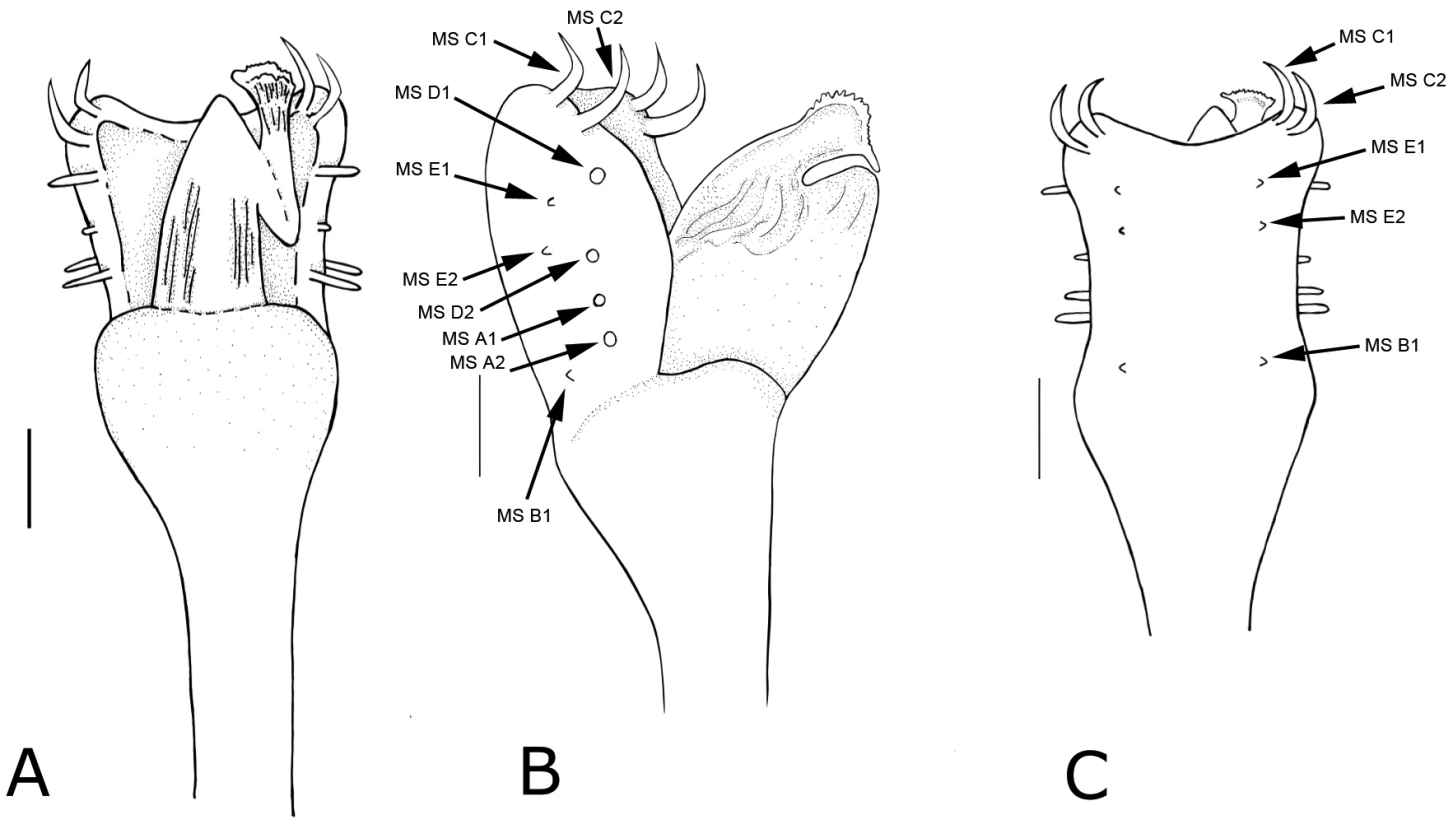
*Platymessa victoriae* sp. n. Penis, distal (male paratype, ICN-AO-1033). **A** Dorsal view **B** Lateral view **C** Ventral view. Locations of paired marginal setae (MS) on the ventral plate are indicated by arrows. MS C1 and C2 occur on the laterodistal margin. MS E1, E2 and B1 occupy the most ventral position and MS D1, D2, A1 and A2 are aligned on the dorsolateral border of the ventral plate. Scale bar: 0.01 mm.

**Measurements: CL**: 1,34mm; DSW: 4,17mm CW 2,31mm; DSL: 4mm; Fe: 1,4; 2,9; 2,6; 3,3 mm. Ti: 1,1; 2,6; 1,5; 1,9 mm. **Dorsum** (Figs 1A, C–D and 2): Dorsal scutum β shaped, body slightly convex posteriorly, cheliceral sockets shallow flanked by subsquare lateral projections and separated by a short triangular median projection. Lateral borders with granules on the protrusion of the dorsal scutum, posterior border with a row of small and scarce granules. Free tergites each with one row of round tubercles. Ocularium with a slight median depression, covered with granules arranged proportionally towards each of the eyes. Dorsal scutum in area I with a pair of relatively large blunt tubercles and many smaller granules; area III with a pair of strongtriangular tubercles that are tilted backwards. Anal operculum with tubercles of medium size. V-shaped color pattern on the cephalic groove and a transverse, discontinuous, substraight line behind the triangular tubercles on area III in the groove between areas III-IV. **Pedipalps** (Fig. 3A–C). Trochanter with a subdistal seta; femur at the dorsal border with a keel and the ventral border with a row of 8 tubercles of variable size; tibia with an ectal laminar projection wider to distal part and in inner side, the projection is smaller and the same size lengthwise; tarsus with scarce setae on the dorsal surface. **Chelicerae.** Basichelicerite with a row of tubercles on the posterior border, the dorsal surface has small granules. Fixed finger with a row of six teeth which decrease in size towards the distal part, moveable finger with a row of 12 small denticles that are equal in size. **Legs.** Coxa IV with a distal prodorsal projection, ventrally with a subdistal blunt tubercle smaller than the prodorsal, c*lavus inguinis* present. Femora of legs I and II substraight with some granules, III and IV slightly curved, in general, densely granulated, with five longitudinal rows of tubercles, the ventral tubercles are larger (Fig. 4A–E); patellae I-IV granulated; tibiae of legs III and IV slightly granulated; tarsi I with basitarsus inflated; tarsal formula: 5 (3); 9-9 (3); 7-7 (3); 7-7 (3). **Genitalia** (Fig. 5A–C). Ventral plate subrectangular, the basal region is narrower than the distal, the lateral margins are subparallel and the distal margin is concave. Truncus apically thickened. On the lateral edges of the distal part of the ventral plate are two pairs of MS - C1 and C2, are strongly curved and flattened; there are two pairs of MS D1 and D2, D1 is well-developed, straight and cylindrical and shorter than C1 and C2 and MS D2 is reduced and cylindrical and occurs dorsally between MS A and C. In the basal part of the ventral plate there are two pairs of MS, A1 and A2 are both well-developed, cylindrical and anchored laterally to the ventral plate, although MS A1 is slightly more dorsal and remains aligned with the MS C1, C2 and D1 and with A2. On the ventral surface are located two pairs of MS, E1 and E2 located at the height of MS D1, both are reduced and aligned with a single pair of MS B1 located ventrally near the apical part of the truncus. On the ventral plate, the microsetae occur in the corners and extend on the lateral margins towards the proximal part of the ventral plate without touching each other. The midfield lacks microsetae. The distribution of microsetae is similar to that observed for *P. h-inscriptum* (Medrano and Kury, 2016).

##### Female allotype

(Fig. 1B). Very similar to the male. Measurements: CL: 1,2mm; DSL: 2,5mm; AW: 1,6mm; DSW: 3,5mm; Fe: 1,4; 2,7; 2,1; 2,7 mm. Ti: 0,9; 2,1; 1,4; 1,9 mm.

##### Sexual dimorphism.

Basitarsus I inflated in males (Fig. 3D), the tubercles of the femora and tibiae of legs III and IV are larger in males than in females, in general the tubercles on legs I-IV are larger in males. The dorsal scutum is almost α shape in males and γ shape in females; c*lavus inguinis* and the body size of females is larger than that of males.

**Table 1.** *Platymessa victoriae* sp. n. Measurements of the legs and pedipalp of the male holotype.

	Leg I	Leg II	Leg III	Leg IV	Pedipalp
**Co**	0,9	1,22	1,42	2,8	0,2
**Tr**	0,4	0,6	0,6	0,8	0,6
**Fe**	1,4	2,9	2,6	3,3	0,7
**Pa**	0,7	1,1	0,9	0,9	0,5
**Ti**	1,1	2,6	1,5	1,9	1,1
**Mt**	1,8	3,5	2,7	3,6	
**Ta**	1,3	2,57	1,6	1,6	0,55

**Table 2.** *Platymessa victoriae* sp. n. Measurements of the legs of the female allotype.

	Leg I	Leg II	Leg III	Leg IV	Pedipalp
**Tr**	0,46	0,68	0,71	0,62	0,66
**Fe**	1,8	3,5	2,73	3,4	1,04
**Pa**	0,6	1,02	0,88	1,02	0,82
**Ti**	1,11	2,86	1,65	2,36	1,09
**Mt**	1,34	3,19	2,19	3,54	
**Ta**	1,14	2,46	1,4	1,62	0,47

##### Variation.

**Figure 6.**
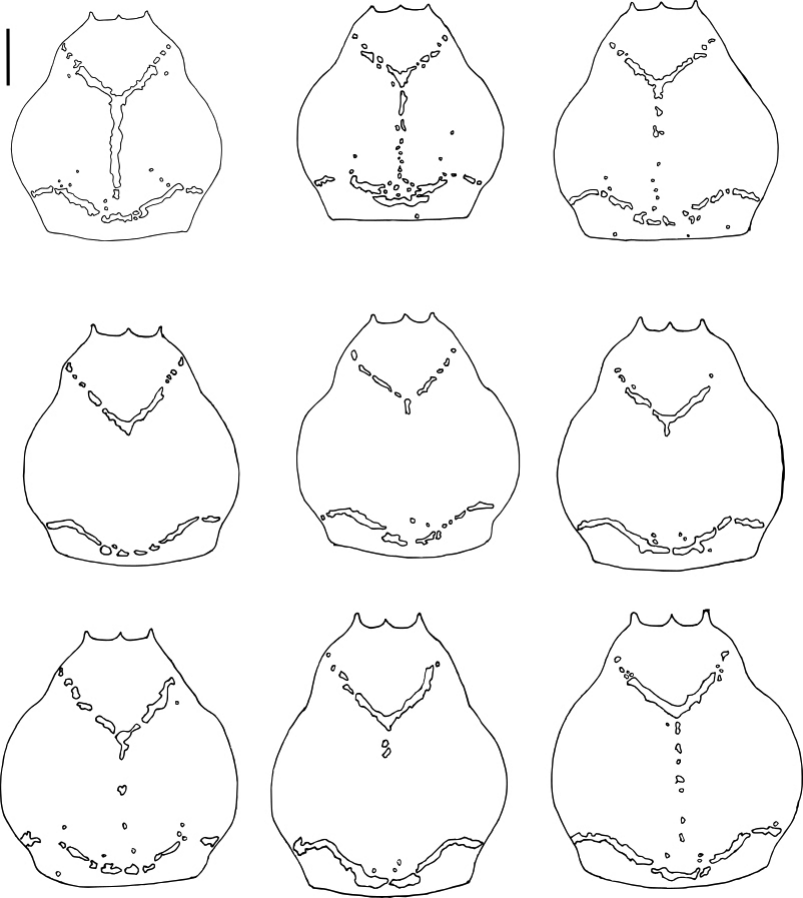
*Platymessa victoriae* sp. n. (male paratypes, ICN-AO-1032, ICN-AO-1034, ICN-AO-1035, ICN-AO-1036, ICN-AO-1037, ICN-AO-1038, ICN-AO-1039, ICN-AO-1040). Intraspecific variation of the pattern of spots in dorsal scutum. Scale bar: 1mm.

The color pattern of dorsal scutum varies especially with respect to the completeness of medial line of the dorsal scutum (Fig. 6).

**Table 3.** Range of measurements of body and appendage in *Platymessa victoriae* sp. n.

	Males n=5			Females n=5		
	Max	Min	Mean	Max	Min	Mean
DSL	5,05	4,19	4,49	4,57	4,12	4,31
DSW	4,80	3,96	4,14	4,06	3,83	3,95
TBL	5,66	4,60	5,01	5,45	4,85	5,11
Fe I	2,02	1,73	1,83	1,76	1,49	1,64
Ti I	1,46	0,82	1,14	1,25	0,95	1,10
Fe II	4,05	3,50	3,72	3,36	3,13	3,27
Ti II	3,23	1,47	2,59	2,64	2,39	2,53
Fe III	3,03	2,68	2,82	2,63	2,34	2,52
Ti III	1,99	1,44	1,65	1,7	1,45	1,59
Fe IV	3,80	3,33	3,55	3,37	3,04	3,22
Ti IV	2,43	2,12	2,33	2,52	1,91	2,28

##### Color in ethanol.

Body is “Cinnamon-Brown” (XV-15’*k*), legs are “Buckthorn-Brown” (XV-17’*i*), blots and lines are “Light Orange-Yellow” (III-17 *d*).

##### Distribution.

Only known from the type locality (Fig. 5).

##### Ecology.

In the Páramo de Sabana Rubia, the temperature may drop to 0 °C, the specimens were collected in the necromass of frailejones (Asteraceae).

**Figure 7.**
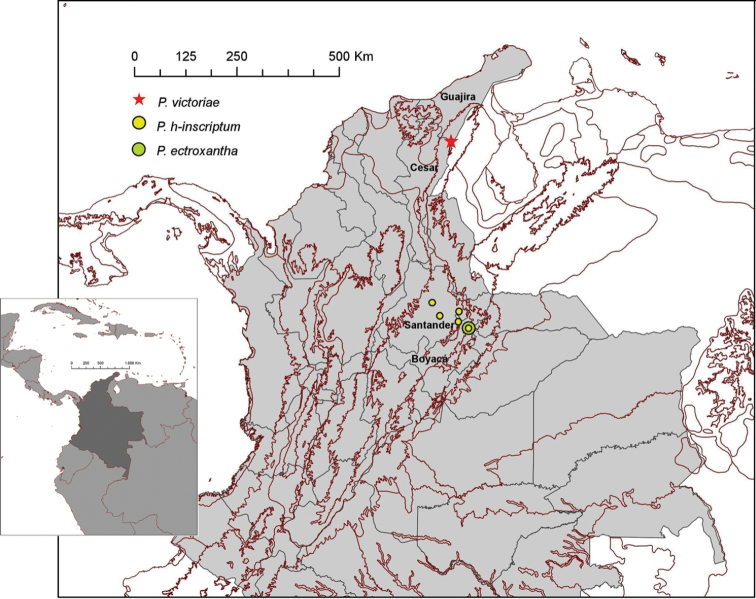
Northwestern region of South America showing the distribution of the three species of *Platymessa* in Colombia. Locations were added using WWF Terrestrial Ecoregions of the World (Olson et al., 2011).

## Discussion

The genus *Platymessa* was revised by Medrano and Kury (2016) and the following combination of characters was used to diagnose the genus: 1) outline of the dorsal scutum β form in males and “almost” α form in females; 2) scutal area V with a pair of small paramedian granules; 3) dorsal color pattern of a “ladder mask”; 4) monomorphic chelicerae, weak with marginal rows of acuminate tubercles of variable size on posterior border; 5) groin warts present, larger in the female; 6) femora III and IV with five longitudinal rows of small tubercles; 7) basitarsomeres of leg I larger than distitarsomeres in males; and 8) cheliceral sockets shallow with lateral triangular projections. *Platymessa victoriae* sp. n. exhibits most of these characteristics except with respect to the armature of the dorsal scutum (scutal area III has a pair of triangular tubercles and scutal area V lacks paired paramedian granules) and the absence of a “ladder mask” with respect to the dorsal coloration.

With regards to penis morphology, the shape of the ventral plate, the number and sizes of the marginal setae (MS), and the distribution of microsetae are similar between *P. h-inscriptum* and *P. victoriae* sp. n. However, there is interspecific variation with respect to the size and position of the MS on the ventral plate. In *P. victoriae* sp. n., MS D1 is more cylindrical and considerably shorter than C1 and C2. In addition, MS A1 and A2 are located more basally on the ventral plate and MS E1 and E2 occur between D1 and D2 (in *P. h-inscriptum*, MS A1 and A2 are more medial and are closer to the margin of the ventral plate and MS E1 and E2 are situated between D1 and A1).

## Supplementary Material

XML Treatment for Platymessa

XML Treatment for Platymessa victoriae

## References

[B1] Kury AB (2016) A classification of the penial microsetae of Gonyleptoidea (Opiliones: Laniatores). Zootaxa 4179: 144–150. https://doi.org/10.11646/zootaxa.4179.1.13 27811701 10.11646/zootaxa.4179.1.13

[B2] Kury AB Pinto-da-Rocha R (2007) Cosmetidae Koch, 1839. In: Pinto-da-Rocha R Machado G Giribet G (Eds) Harvestmen: The Biology of the Opiliones. Harvard University Press, Cambridge and London, 182–185.

[B3] Kury AB Villarreal O Sampaio C (2007) Redescription of the type species of *Cynorta* (Arachnida, Opiliones, Cosmetidae). Journal of Arachnology 35: 325–333. https://doi.org/10.1636/H06-35.1

[B4] Kury AB Villarreal O (2015) The prickly blade mapped: Establishing homologies and a chaetotaxy for macrosetae of penis ventral plate in Gonyleptoidea (Arachnida, Opiliones, Laniatores). Zoological Journal of the Linnean Society 174: 1–46. https://doi.org/10.1111/zoj.12225

[B5] Medrano M Kury AB (2016) Characterization of *Platymessa* with redescription of the type species and a new generic synonymy (Arachnida, Opiliones, Cosmetidae). Zootaxa 4085: 52–62. http://doi.org/10.11646/zootaxa.4085.1.2 27394288 10.11646/zootaxa.4085.1.2

[B6] de Mello-LeitãoLeitão CF (1941) Alguns opiliões novos da Colombia. Anais da Academia Brasileira de Ciências 13: 165–171.

[B7] Olson DM Dinerstein E Wikramanayake ED Burgess ND Powell GVN Underwood EC D’amico JA Itoua I Strand HE Morrison JC Loucks CJ Allnutt TF Ricketts TH Kura Y Lamoreux JF Wettengel W Hedao P Kassem KR (2001) Terrestrial Ecoregions of the World: A New Map of Life on Earth. BioScience 51: 933–938. http://dx.doi.org/10.1641/0006-3568(2001)051[0933:TEOTWA]2.0.CO;2

[B8] Pinto-da-Rocha R Hara MR (2011) Redescription of *Platygyndes* Roewer 1943, a false Gonyleptidae, (Arachnida, Opiliones, Cosmetidae). ZooKeys 143: 1–12. https://doi.org/10.3897/zookeys.143.1916 10.3897/zookeys.143.1916 PMC3208530 22144863

[B9] Ridgway R (1912) Colors standards and color nomenclature. A. Hoen & Company, Washington, DC, 44 pp.

[B10] Roewer CF (1963) Opiliones aus Peru und Colombien. [Arachnida Arthrogastra aus Peru V]. Senckenbergiana Biologica 44: 5–72.

